# Psychometric Properties of the Perceived Stress Questionnaire (PSQ) in 15–16 Years Old Norwegian Adolescents

**DOI:** 10.3389/fpsyg.2018.01850

**Published:** 2018-10-01

**Authors:** Berit Østerås, Hermundur Sigmundsson, Monika Haga

**Affiliations:** ^1^Department of Neuromedicine and Movement Science (INB), Norwegian University of Science and Technology, Trondheim, Norway; ^2^Department of Psychology, Norwegian University of Science and Technology, Trondheim, Norway

**Keywords:** adolescent stress, psychosomatic, perceived stress questionnaire, psychometric properties, construct validity, invariance

## Abstract

A better understanding about prerequisites of health and well-being in adolescents is important to prevent chronicity and comorbidities of stress and to improve health promotion in this group. For this purpose, useful instruments are required. The Perceived Stress Questionnaire (PSQ) is developed for research, with an emphasis on predictive validity. The PSQ comprises different components of stress, and the instrument might be useful in studying prerequisites and predictors of health and well-being in adolescents. However, the instrument has not been evaluated in Norwegian psychosomatic populations and in adolescents. Moreover, the factor structure of the PSQ seems to vary between populations, and invariance across gender and concurrent validity regarding mindfulness are not previously tested. The objective of this study were to evaluate the psychometric properties of the Norwegian version of the PSQ in adolescents, including evaluate the fit of previously proposed PSQ-models in females and males and test measurement invariance across gender. Concurrent validity with respect to mindfulness (Mindful Attention Awareness Scale- Adolescent [MAAS-A]) was preliminary evaluated. Confirmatory factor analysis for each previously proposed model was conducted, separately for females and males. Multi-group factor analyses were performed to test measurement invariance of the different PSQ-models across gender. The associations between the PSQ and the MAAS-A and inter-scale correlations were preliminary evaluated. Preceding the data collection and main analyses, the instruments were translated to Norwegian following standardized procedures. The participants in study were Norwegian adolescents aged 15–16 years (*N* = 524). The overall PSQ performance seemed to correspond to previous findings, and internal consistency was supported across gender. A four-factor model of the PSQ showed best fit to the data in both females and males and configural and metric invariance seemed supported. Full scalar invariance was not supported for the four-factor model, implying that cross-group comparisons (between females and males) on latent means may be uncertain and must be interpreted with caution. Concurrent validity with respect to mindfulness (MAAS-A) was preliminary supported. Further studies might be needed to confirm the findings from this study.

## Introduction

We have insufficient understanding about the prerequisites of health and well-being in adolescents and its development, and how stress might influence and predict this development (Moreira et al., 2015; Schulz and Vögele, 2015; Martín-María et al., 2016; Moksnes and Espnes, 2017). Apparently, there is an increase of stress-related symptoms and diseases among young people, including long-term pain (Lager et al., 2012; Wiklund et al., 2012; Eckhoff and Kvernmo, 2014; Skrove et al., 2015; Mikkonen et al., 2016). Persistent stress might cause impaired function and disability on a longer term (Cohen et al., 2007; Chapman et al., 2008; Rampton, 2011; Schraml et al., 2011; Peters et al., 2017). To increase our insight into adolescent health and well-being, and to improve health promotion in this group, appropriate instruments are required. This involves useful stress-instruments with a potential to predict psychosomatic health in adolescents (Schraml et al., 2011; Moksnes and Espnes, 2017).

Stress is experienced when a person perceives that the demands overload or exceed the personal and social resources the individual is able to mobilize, according to a traditional transactional model of stress (Lazarus and Folkman, 1984). In this view, neither the environmental event nor the persons' response defines stress, rather the individuals perception of the situation is the critical factor. The modified transactional model of stress explains how several factors contribute in the stress perception process, involving personal aspects, stress exposures, and reactions (Levenstein et al., 1993; Kocalevent et al., 2007). Health consequences to stress depends on the individual appraisal of available resources under the influence of personality characteristics, according to the modified transactional model of stress (Levenstein et al., 1993; Kocalevent et al., 2007). Stress might be positive or “good,” as it provides energy and may enhance coping in demanding and challenging situations, but might also be detrimental and disabling, particularly at the longer term (Rampton, 2011; Peters et al., 2017).

There are several instruments aiming at assessing stress in young people, which are developed on different theoretical and clinical backgrounds and for different purposes. The Kessler six-item psychological distress (K6) scale, for example, is a short scale that screens for serious mental illness or emotional disturbance (Kessler et al., 2002; Green et al., 2010; Peiper et al., 2015, 2016). The Adolescent Stress Questionnaire (ASQ) is a 56-item instrument, focusing on 10 different adolescent-specific stress domains, which has demonstrated negative associations with adolescent emotional symptoms, self-esteem and self-efficacy scores (Byrne et al., 2007; McKay et al., 2016). By use of a 30-item Norwegian version of the instrument (ASQ-N) (Moksnes and Espnes, 2011), Moksnes and Espnes (2017) recently found associations between the scale score and subjective health complaints, in terms of a combination of somatic and mental symptoms.

The Perceived Stress Questionnaire (PSQ) is developed for clinical psychosomatic research, with a particular emphasis on its predictive validity regarding development of stress-related disorders (Levenstein et al., 1993, 2000; Sanz-Carrillo et al., 2002; Fliege et al., 2005). This is relevant in the endeavor to increase our understanding about prerequisites and predictors of health and well-being in young people. The PSQ permits assessments of subjective experiences of perceived stressful situations and stress reactions, emphasizing cognitive perceptions more than emotional states or specific life events, and it is considered valid in the context of a transactional view of stress (Kocalevent et al., 2007). The general form of the instruction asks questions related to “the last two years” and the recent form asks about situations taking place “during the last four weeks,” addressing chronic and acute experience with stressful events and activities (Levenstein et al., 1993; Montero-Marin et al., 2014a). Originally designed in English, this instrument has been translated into Italian, German, Spanish, and Swedish and validated in populations of psychiatric inpatients and outpatients, students, health workers, psychosomatic patients, and healthy adults (Levenstein et al., 1993; Bergdahl and Bergdahl, 2002; Sanz-Carrillo et al., 2002; Fliege et al., 2005). Psychometric evaluation of a Norwegian version of the instrument is missing.

Previous studies have supported aspects of construct validity and test-retest reliability of the PSQ (Levenstein et al., 1993, 2000; Sanz-Carrillo et al., 2002; Fliege et al., 2005; Kocalevent et al., 2007; Montero-Marin et al., 2014a). Internal consistency (measured by Cronbach's alpha [α]) of the PSQ has ranged from 0.85 to 0.93 (Levenstein et al., 1993; Kocalevent et al., 2007). Test-retest reliability (measured by Pearson correlation coefficients [r]) has ranged between 0.80 and 0.86 (Levenstein et al., 1993; Sanz-Carrillo et al., 2002). Construct/concurrent validity of the PSQ has been demonstrated in terms of positive associations with compatible stress measures, including The Trier Inventory for the Assessment of Chronic Stress (TICS) (Schulz and Schlotz, 1999) and Cohen's Perceived Stress Scale (PSS) (Cohen et al., 1983). Additionally, PSQ appears positively associated with anxiety and depression, neuroticism, burnout (in terms of exhaustion, cynicism, and lack of efficacy), and somatic symptoms (Levenstein et al., 1993; Sanz-Carrillo et al., 2002; Fliege et al., 2005). Furthermore, PSQ appears to be negatively associated with reliance, self-efficacy, optimism, and health-related quality of life (Fliege et al., 2005; Kocalevent et al., 2007; Montero-Marin et al., 2014a,b). As a measure of criterion validity, Fliege et al. (2005) demonstrated that higher perceived stress scores (PSQ) was associated with indicators of immunological imbalance in women who have had a miscarriage. Moreover, PSQ seem to differ between patients and healthy individuals and seems sensitive to change after treatment (Fliege et al., 2005). Finally, the PSQ has demonstrated good predictive validity for stress-related diseases including ulcerative colitis (Levenstein et al., 2000).

The initial psychometric study of the 30-item PSQ was based on relatively small samples (*N* = 230) of English and Italian patients, students and health workers (Levenstein et al., 1993). Seven factors were extracted by principal components analyses (*N* = 230); harassment (4 items), irritability (2 items), lack of joy (7 items), fatigue (4 items), worries (5 items), tension (4 items), and overload (4 items). In later psychometric studies, larger samples have been included and a smaller number of factors extracted and confirmed. Fliege et al. (2005) confirmed a four-factor solution of a 20-item German version of the PSQ, based on exploratory (*N* = 650) and confirmatory analyses (*N* = 1,808) in populations of patients, healthy adults and medical students. Exploratory principal component factor analysis was used on data from the first sample (*N* = 650), while linear structural equation modeling (SEM, Program Amos) and multi-sample analyses were conducted on data from the second sample (*N* = 1,808). Four factors with structural equality (all comprising 5 items) were confirmed, including worries (α = 0.77 in students), tension (α = 0.83 in students), (lack of) joy (α = 0.82 in students), and demands (α = 0.81 in students) (Fliege et al., 2005). Montero-Marin et al. (2014a) reassessed the psychometric characteristics of the PSQ in a sample of dental students (*N* = 314), extracting a two-factor solution of the instrument. Polychoric correlation matrix was used due to non-normal distribution of scores. Parallel analysis and confirmatory factor analyses (applying unweighted least squares from a polychoric matrix) were applied on data from the same sample (*N* = 314), as well as item-reduction based on Item Response Theory (IRT)-discrimination. The two factors extracted were frustration (12 items) and tenseness (12 items) (Montero-Marin et al., 2014a).

Different PSQ-factors have been associated with health complaints in young people, including fatigue (Kocalevent et al., 2011) and burnout subtypes (Montero-Marin et al., 2014b). Albeit PSQ seems to be a valid and reliable instrument for assessing perceived stress, also in young adults (Fliege et al., 2005; Stinson et al., 2010; Montero-Marin et al., 2014a), there is lacking psychometric evaluation of this instrument in adolescents. This includes measurement invariance across gender and concurrent validity with respect to mindfulness. Measurement invariance across gender is important to investigate, checking that the same construct is measured in females and males, as there are plausible gender differences in stress perception and reactions, also in adolescents (Sarrasin et al., 2014; Mayor, 2015). Concurrent validity of the PSQ with respect to mindfulness is relevant to evaluate, as mindfulness is considered to be negatively associated with stress (Brown et al., 2007; Kabat-Zinn, 2011), also in adolescents (Brown et al., 2011; Galla, 2016). Mindfulness is presumed to prevent stress and negative effects of stress and beneficially influence health and well-being in adolescents (Zoogman et al., 2015; Bamber and Kraenzle Schneider, 2016; Bluth et al., 2016; Galla, 2016; Sibinga et al., 2016).

A relevant scale for adolescents is the Mindful Attention Awareness Scale Adolescent (MAAS-A), which is one of the most commonly applied instruments for assessing adolescent mindfulness (Brown et al., 2011; de Bruin et al., 2011). Previous studies have demonstrated that the MAAS-A is valid and reliable in adolescent populations, providing support of the single-factor structure of the instrument, internal consistency, convergent and divergent validity, and test–retest reliability (Brown et al., 2011; de Bruin et al., 2011). According to the test-authors, the MAAS-A aims to assess mindfulness in day-to-day life, which involves a receptive state of attention that, informed by an awareness of present experience, simply observes what is taking place (Brown and Ryan, 2003; Brown et al., 2007). The authors have recently specified that the MAAS or MAAS-A focus on a quality of attentiveness involved in mindfulness (Quaglia et al., 2016). In young people, high trait mindfulness measured by the MAAS has shown to be preventive of a psychological stress response (but not salivary cortisol reaction) to social evaluative stress (Trier Social Stress Test [TSST]) (Creswell et al., 2014) and to accompany reduction of stress, anxiety and depression (succeeding a mindfulness-based stress reduction program) (Song and Lindquist, 2015). Recently, a study in Norwegian adolescents demonstrated negative association between stress (school-related) and the MAAS-A (validated short-form) (Smith et al., 2017).

The PSQ might be useful in studying and understanding prerequisites and predictors of health and well-being in adolescents. However, there is lacking psychometric evaluation of the PSQ in adolescents and in Norwegian populations. Moreover, the factor structure of the PSQ seems to vary between populations (Fliege et al., 2005), and measurement invariance across gender and concurrent validity with respect to mindfulness are previously not tested. The objective of this study was to evaluate the psychometric properties of the Norwegian version of the PSQ in adolescents, including evaluate the fit of previously proposed PSQ-models in females and males and test measurement invariance across gender. Additionally, concurrent validity with respect to mindfulness was preliminary evaluated.

## Materials and methods

### Participants and design

The cross-sectional sample of this study includes Norwegian pupils in 10th grade (15 or 16 years), recruited from public schools in the Trondheim municipality in Norway. Out of 17 invited schools, seven agreed to participate. The schools varied in size and localization (from city to suburb), including pupils with different sociocultural and economic backgrounds, considered to provide a representative sample of 15–16 years old adolescents from this region. Five hundred and forty questionnaires were distributed. The total number of completed questionnaires returned was *N* = 524, giving an overall response rate of 97 per cent. The sample comprised 280 (53.4 %) females and 244 (46.6 %) males, considered adequate for analyses (Muthén and Muthén, 2002; Kline, 2011). The data collection period was from spring 2013 to autumn 2015.

### Study measurements

#### The perceived stress questionnaire (PSQ)

The recent form of the 30-item PSQ, which is used in this work, refer to the period of the last 4 weeks and can be answered with a four-point Likert-type scale (1 = almost never, 2 = sometimes, 3 = often, 4 = usually) (Levenstein et al., 1993; Fliege et al., 2005; Kocalevent et al., 2007). Higher scores indicate more severe perceived stress. The resulting PSQ total score is linearly transformed between zero and one; PSQ = (raw value−30)/90 (Levenstein et al., 1993).

The authors of the PSQ granted us permission to translate and back translate the PSQ and authorized our final version. First, two independent bilingual, native Norwegian translators translated the questionnaire from English to Norwegian. Then the two versions were compared and differences were addressed and adjusted to attain the most appropriate item wordings. Two other bilingual translators did the back-translation from Norwegian to English. One of them was native Norwegian and the other a native English speaker and both were unfamiliar with the original PSQ. The original and back-translated versions of the questionnaire were compared to ensure equality. The test authors authorized the back-translated version. The provisional forward translation was then pilot-tested, screening the feasibility of the instrument in the target population (Norwegian adolescents aged 15–16 years) in terms of if the questions and scoring procedures seemed understandable and manageable to the adolescents. A convenience sample of ten 15–16 year olds (five females and five males) was included (Connelly, 2008). All adolescents completed the questionnaires within a reasonable time and no one needed assistance. There were no missing values and the average item and scale scores corresponded to previous findings in young populations (Fliege et al., 2005).

#### The mindful attention awareness scale for adolescents (MAAS-A)

MAAS-A includes 14 items where the responses are made on a 6-point scale (1 = almost always, 2 = very frequently, 3 = somewhat frequently, 4 = somewhat infrequently, 5 = very infrequently, 6 = almost never) (Brown et al., 2011). Higher scores reflect higher trait mindfulness, and the total sum score of the MAAS-A is the computed mean of the item scores (Brown et al., 2011). The authors of the MAAS-A granted us permission for translation and back translation of the MAAS-A, and authorized our final version. The translation process was similar to the one described for the PSQ.

### Ethics and procedures

The Regional Committee for Medical Research Ethics in Trondheim approved the data collection processes and the entire study, and the study was in line with the Declaration of Helsinki (The World Medical Association (WMA), 2013). The purpose of the study, the outcome measures and the procedures were explained and in consensus with the participating school (principal and teachers). The adolescents and the parents received an information letter that briefly explained the purpose of the study. In all stages of the data collection, it was emphasized that participation was voluntary, anonymous, and confidential, and that the participants were free to withdraw from the study at any point without giving a reason. Informed consent was obtained from all participants. Students already 16 years were responding on their own behalf. Students not yet turned 16 at the time of data collection received a written consent from their parents. Participating in the study was not presumed to affect the students in any negative way.

### Statistical analysis

The statistical analyses were conducted in IBM SPSS 25 and Amos version 25. Preliminary analyses were performed to check for univariate and multivariate normality. Descriptive analyses including means, standard deviations, skewness and kurtosis, and corrected item-scale correlations were calculated to evaluate the performance of the PSQ items, also separately for gender. Cronbach's alpha (α) reliability coefficients were computed to measure internal consistency of the scales.

Kaiser-Meyer-Olkin (KMO) statistics of the 30-item PSQ was conducted to measure the quality of the correlation matrix, as well as the Bartlett test of sphericity for measuring sampling adequacy. To evaluate the fit of the previously proposed PSQ-models, confirmatory factor analysis (CFA) was used. We specified a CFA for each previously proposed model, i.e., one-factor, two-factor, four-factor, and seven-factor, of the PSQ, reflecting the theoretically operationalization/ dimensionality of the construct. The CFA-models were fitted for each group separately, i.e., for females and males. A combination of absolute, relative, and non-centrality-based fit indices were used collectively to indicate the global fit of the models to the data: the chi-square model test, the Akaike information criterion (AIC), the non-normed fit index (NNFI), the comparative fit index (CFI), the root mean square error of approximation (RMSEA), and the standardized root mean square residual (SRMR). CFI and NNFI should be close to 0.95, the RMSEA should be close to 0.06, and the SRMR should be < 0.10 (Hu and Bentler, 1999) to conclude with high model fit between the hypothesized model and the observed data. Hu and Bentler (1999) noted that a cutoff value greater than 0.90 for the CFI and NNFI is required to reject adequate proportions of miss-specified models. However, these criteria are merely guidelines, i.e., if previous models generate CFI values of 0.70 a CFI value of 0.85 represents progress and thus should be acceptable (Bollen, 1989). The AIC adjust for model complexity and was used as a measure of model parsimony to compare the fit of the models; the lower the value, the better the fit. Although the chi-square (χ2) test is reported, this was not used as a primary measure of model fit because of its sensitivity to sample size, high correlations between variables, and variables with high proportions of unique variance (Kline, 2011; Gu et al., 2016).

Multi-group factor analyses were performed to test measurement invariance of the different PSQ-models across gender. To test for configural invariance, i.e., whether the same CFA was valid in both females and males, no constrains were applied. This level of invariance tests whether an equivalent number of factors are extracted across groups and whether the same items load on each factor across groups (van de Schoot et al., 2012). Metric invariance was tested by constraining factor loadings. This level of invariance tests whether respondents across groups attribute the same meaning to the latent construct, i.e., if both groups (females and males) interpret the items in the same way. A lack of metric invariance may suggest that some items are either more ambiguous or less important for one group than for another (Campbell et al., 2008). Scalar (or strong) invariance was tested by constraining both factor loadings and intercepts. This level of invariance implies that the meaning of the construct (the factor loadings) and the levels/thresholds of the underlying items (intercepts) are equal in both groups, indicating that the groups use the response scale in a similar way (Lavoie and Douglas, 2012). For straightforward interpretation of latent means and correlations across groups, both the factor loadings and intercepts should be the same across groups, i.e., scalar invariance should be supported (van de Schoot et al., 2012). Error (or strict) invariance was tested by additionally constraining the residual variances. This strict or full uniqueness measurement invariance means that the explained variance for every item is the same across groups, i.e., the latent construct is measured identically across groups. If error variances are not equal, groups can still be compared on the latent variable, but this is measured with different amounts of error between groups (van de Schoot et al., 2012). Measurement invariance was evaluated by change in fit to the previous, less stringent model: change in CFI (ΔCFI) ≤ 0.01, change in NNFI (ΔNNFI) ≤ 0.02, and change in RMSEA (ΔRMSEA) ≤ 0.015 indicated support for the more stringent model (Cheung and Rensvold, 2002; Chen, 2007; Schnabel et al., 2015; Gu et al., 2016).

To investigate inter-scale correlations and the associations between the PSQ and the MAAS-A, Pearson correlation coefficients (r) were used. The analyses involving MAAS-A were based on data from a smaller sample (*n* = 101, one school), as the more comprehensive survey (comprising both the PSQ and the MAAS-A) was only approved for one of the schools.

## Results

Descriptives of the PSQ-items are presented in Table 1. The PSQ scores were in general normally distributed, as assessed by checking histograms, box plots, skewness, and kurtosis values. Skewness and kurtosis of item scores were as following; −0.258 and −0.553 (item 1), 0.0370 and −0.361 (item 2), 0.709 and −0.006 (item 3), 0.307 and −0.464 (item 4), 1.681 and 2.186 (item 5), 1.272 and 1.001 (item 6), 0.181 and −0.861 (item 7), 0.370 and −0.605 (item 8), 0.427 and −0.547 (item 9), 0.131 and −0.704 (item 10), 0.702 and 0.061 (item 11), 0.788 and −0.014 (item 12), −0.080 and −0.609 (item 13), 0.880 and 0.320 (item 14), 1.084 and 0.270 (item 15), 0.351 and −0.579 (item 16), 0.828 and −0.211 (item 17), 0.689 and −0.391 (item 18), 0.826 and −0.175 (item 19), 1.211 and 1.129 (item 20), 0.795 and 0.022 (item 21), 0.583 and −0.604 (item 22), 0.522 and −0.345 (item 23), 0.956 and −0.066 (item 24), 0.300 and −0.573 (item 25), 0.806 and −0.200 (item 26), 1.083 and 0.468 (item 27), 0.833 and 0.131 (item 28), 0.277 and −0.885 (item 29), and 0.391 and −0.809 (item 30), respectively. Except from item 5, 6, and 20, which are omitted in the four-factor model, all values were within recommended range (George and Mallery, 2010; Kerman and McDonald, 2013).

**Table 1:** PSQ item mean (standard deviation) scores and corrected item-scale correlations, overall, and separately for gender.

	**Overall (*****N*** = **524)**		**Females (*****n*** = **280)**		**Males (*****n*** = **244)**	
**Items**	**Mean (SD)**	**Item-scale**	**Mean (SD)**	**Item-scale**	**Mean (SD)**	**Item-scale**
1. You feel rested (r)	2.53 (0.84)	0.38^**^	2.57 (0.85)	0.50^**^	2.48 (0.83)	0.19^**^
2. You feel that too many demands are being made on you	2.24 (0.84)	0.53^**^	2.33 (0.84)	0.59^**^	2.15 (0.82)	0.43^**^
3. You are irritable or grouchy	1.71 (0.72)	0.47^**^	1.82 (0.73)	0.49^**^	1.59 (0.69)	0.39^**^
4. You have too many things to do	2.29 (0.85)	0.51^**^	2.43 (0.88)	0.57^**^	2.13 (0.79)	0.36^**^
5. You feel lonely or isolated	1.48 (0.78)	0.57^**^	1.61 (0.71)	0.57^**^	1.32 (0.63)	0.50^**^
6. You find yourself in situations of conflict	1.51 (0.71)	0.46^**^	1.54 (0.71)	0.48^**^	1.47 (0.71)	0.45^**^
7. You feel you're doing things you really like (r)	2.12 (0.85)	0.42^**^	2.27 (0.84)	0.41^**^	1.94 (0.83)	0.38^**^
8. You feel tired	2.54 (0.82)	0.52^**^	2.62 (0.81)	0.61^**^	2.45 (0.82)	0.37^**^
9. You fear you may not manage to attain your goals	2.19 (0.90)	0.58^**^	2.38 (0.89)	0.59^**^	1.98 (0.88)	0.52^**^
10. You feel calm (r)	2.31 (0.87)	0.41^**^	2.47 (0.83)	0.43^**^	2.12 (0.89)	0.32^**^
11. You have too many decisions to make	1.89 (0.81)	0.54^**^	2.0 (0.82)	0.49^**^	1.77 (0.79)	0.59^**^
12. You feel frustrated	1.83 (0.84)	0.68^**^	2.02 (0.86)	0.69^**^	1.62 (0.76)	0.61^**^
13. You are full of energy (r)	2.48 (0.85)	0.37^**^	2.60 (0.85)	0.42^**^	2.34 (0.82)	0.25^**^
14. You feel tense	1.77 (0.80)	0.50^**^	1.82 (0.84)	0.55^**^	1.72 (0.75)	0.42^**^
15. Your problems seem to be piling up	1.70 (0.88)	0.66^**^	1.80 (0.92)	0.70^**^	1.59 (0.82)	0.60^**^
16. You feel you're in a hurry	2.19 (0.88)	0.56^**^	2.38 (0.88)	0.57^**^	1.96 (0.83)	0.48^**^
17. You feel safe and protected (r)	1.85 (0.91)	0.46^**^	1.92 (0.92)	0.49^**^	1.77 (0.89)	0.41^**^
18. You have many worries	1.99 (0.93)	0.67^**^	2.21 (0.98)	0.71^**^	1.73 (0.81)	0.53^**^
19. You are under pressure from other people	1.83 (0.89)	0.58^**^	2.01 (0.96)	0.59^**^	1.61 (0.74)	0.50^**^
20. You feel discouraged	1.61 (0.77)	0.55^**^	1.75 (0.83)	0.57^**^	1.46 (0.66)	0.44^**^
21. You enjoy yourself (r)	1.80 (0.81)	0.51^**^	1.88 (0.83)	0.54^**^	1.70 (0.79)	0.45^**^
22. You are afraid for the future	2.05 (0.96)	0.53^**^	2.29 (0.95)	0.49^**^	1.78 (0.89)	0.53^**^
23. You feel you're doing things because you have to (…)	2.10 (0.87)	0.52^**^	2.17 (0.88)	0.61^**^	2.01 (0.86)	0.39^**^
24. You feel criticized or judged	1.80 (0.93)	0.64^**^	1.91 (1.0)	0.66^**^	1.67 (0.82)	0.59^**^
25. You are lighthearted (r)	2.25 (0.87)	0.30^**^	2.23 (0.83)	0.36^**^	2.27 (0.92)	0.26^**^
26. You feel mentally exhausted	1.90 (0.92)	0.63^**^	2.04 (0.97)	0.70^**^	1.75 (0.84)	0.48^**^
27. You have trouble relaxing	1.73 (0.86)	0.60^**^	1.85 (0.92)	0.63^**^	1.58 (0.77)	0.52^**^
28. You feel loaded down with responsibility	1.77 (0.80)	0.59^**^	1.85 (0.84)	0.64^**^	1.68 (0.75)	0.50^**^
29. You have enough time for yourself (r)	2.16 (0.93)	0.40^**^	2.25 (0.96)	0.41^**^	2.05 (0.88)	0.37^**^
30. You feel under pressure from deadlines	2.31 (0.97)	0.56^**^	2.39 (1.0)	0.61^**^	2.22 (0.93)	0.48^**^
Total PSQ-score	0.33 (0.16)	-	0.37 (0.18)		0.29 (0.13)	
Cronbach's α	0.93		0.94		0.89	

PSQ, Perceived Stress Questionnaire; r, reversed;

** *p < 0.01*.

The overall PSQ mean (SD) score was 0.33 (0.16), and the internal consistency as measured by Cronbach's α was 0.93. Cronbach's α for the MAAS-A (based on *n* = 101) was 0.84. For females, the PSQ mean (SD) score was 0.37 (0.18) with a Cronbach's α of 0.94. For males, mean (SD) PSQ score was 0.29 (0.13), and internal consistency as measured by Cronbach's α was 0.89 (Table 1). The highest mean scores for both genders appeared for item 1 (“You feel rested,” reversed index), item 8 (“You are tired”), and for item 13 (“You are full of energy”, reversed index). In general, the mean PSQ scores were highest in females, besides for item 25 (“You feel lighthearted,” reversed index), which was highest in males (Table 1). The corrected item-scale correlations were also in general higher in females (Table 1).

The Kaiser-Meyer-Olkin measure of the quality of the correlation matrix for the overall PSQ was high (KMO = 0.94), and a significant Bartlett test of sphericity justified a dimension reducing procedure such as the factor analysis. The measure of sampling adequacy was over 0.80, so the items could be considered apt for factor analyses. Two CFA's were conducted for each model of the PSQ, separately for females and males. The models were modified by covarying error terms with large shared variance. The four-factor model demonstrated best fit to the data for both females and males (Table 2). In males, the NNFI-value was in the lower bound but represented a progress compared to the other models (Bollen, 1989; Hu and Bentler, 1999), and the other fit-indices suggested acceptable fit (Table 2). Hence, we proceeded to the next level of analyses.

**Table 2:** Model fit and measurement invariance tests across gender for the Norwegian version of the PSQ, according to the one-factor, two-factor, four-factor, and seven-factor model, *N* = 524.

**Model**		**CFI**	**RMSEA (90% CI)**	**NNFI**	**SRMR**	**χ^2^**	**Df**	**AIC**	**ΔCFI**	**ΔNNFI**	**ΔRMSEA**
PSQ one-factor	Females	0.910	0.054 (0.047–0.060)	0.895	0.041	680.661	375	860.661			
	Males	0.896	0.045 (0.037–0.053)	0.879	0.037	562.216	375	742.216			
	Configural	0.905	0.035 (0.032–0.039)	0.890	0.039	1242.869	750	1602.869			
	Metric	0.899	0.036 (0.033–0.039)	0.887					0.006	0.003	0.001
	Scalar	0.881	0.038 (0.035–0.041)	0.873					0.018	0.014	0.002
	Error factorial	0.873	0.038 (0.035–0.041)	0.873					0.008	0.000	0.000
PSQ two-factor	Females	0.924	0.054 (0.046–0.062)	0.911	0.039	430.740	237	556.740			
	Males	0.897	0.047 (0.038–0.057)	0.880	0.037	365.897	237	491.897			
	Configural	0.915	0.036 (0.032–0.040)	0.901	0.038	796.634	474	1048.634			
	Metric	0.909	0.037 (0.032–0.041)	0.899					0.006	0.002	0.001
	Scalar	0.889	0.039 (0.035–0.043)	0.883					0.020	0.016	0.002
	Error factorial	0.878	0.040 (0.036–0.044)	0.880					0.011	0.003	0.001
PSQ four-factor	Females	0.927	0.057 (0.047–0.067)	0.913	0.043	305.158	160	405.158			
	Males	0.914	0.046 (0.034–0.057)	0.897	0.039	242.017	160	342.017			
	Configural	0.922	0.037 (0.032–0.042)	0.908	0.041	547.168	320	747.168			
	Metric	0.917	0.037 (0.032–0.042)	0.907					0.005	0.001	0.000
	Scalar	0.885	0.042 (0.038–0.047)	0.879					0.032	0.028	0.005
	Error factorial	0.882	0.041 (0.037–0.046)	0.885					0.003	−0.006	−0.001
PSQ seven-factor	Females	0.853	0.069 (0.063–0.075)	0.830	0.046	874.968	376	1052.968			
	Males	0.841	0.056 (0.049–0.063)	0.816	0.040	661.814	376	839.814			
	Configural	0.849	0.045 (0.042–0.048)	0.825	0.043	1536.754	752	1892.754			
	Metric	0.841	0.045 (0.042–0.048)	0.823					0.008	0.002	0.000
	Scalar	0.823	0.047 (0.043–0.050)	0.811					0.018	0.012	0.002
	Error factorial	0.811	0.046 (0.044–0.049)	0.811					0.012	0.000	−0.001

*PSQ, Perceived Stress Questionnaire; AIC, Akaike information criterion; CFI, comparative fit index; CI, confidence interval; NNFI, non-normed fit index; RMSEA, root mean square error of approximation; SRMR, standardized root mean square residual; χ2, chi-square*.

Next, we tested for measurement invariance with equality constraints in multi-group analyses; see Table 2 for the fit indices. The four-factor model showed the lowest AIC value and therefore the best trade-off between model fit and model complexity. The other fit indices also suggested best fit for the four-factor model. Acceptable changes in fit (ΔCFI, ΔNNFI and ΔRMSEA) to the previous less stringent model suggested support for configural and metric invariance for the four-factor model (Table 2). When constraining both factor loadings and intercepts, the change in fit (with respect to ΔCFI and ΔNNFI) to the previous less stringent model exceeded the recommended range. Hence, full scalar invariance seemed not supported for the four-factor model. For the record, a summary of complete invariance analyses (including error or strict invariance) is reported for all models with respect to the CFI, NNFI and RMSEA despite the preceding level of factorial invariance was not clearly supported.

Correlations (r) between the overall PSQ and the subscales from the four-factor model (worries, tension, [lack of] joy, and demands) are presented in Table 3, showing strongest factor-scale association for “worries” (0.88) and strongest inter-factor association between “worries” and “demands” (0.65). The lowest factor-scale association appeared for “joy” (0.69), and the lowest inter-factor association appeared between “joy” and “demands” (0.40) (Table 3). The associations between the PSQ (overall and subscales from the four-factor model) and MAAS-A are also presented in Table 3, showing the strongest negative correlation (*p* < 0.01) between the MAAS-A and overall PSQ (−0.59) and tension (−0.52).

**Table 3:** Associations (r) between the PSQ, the subscales, and the MAAS-A.

	**PSQ Overall**	**Worries**	**Tension**	**Joy**	**Demands**	**MAAS-A**
PSQ overall	–					
Worries	0.88^**^	–				
Tension	0.82^**^	0.64^**^	–			
Joy	0.69^**^	0.49^**^	0.51^**^	–		
Demands	0.80^**^	0.65^**^	0.61^**^	0.40^**^	–	
MAAS-A	−0.59^**^	−0.49^**^	−0.52^**^	−0.35^**^	−0.48^**^	–

*MAAS-A, Mindful Attention Awareness Scale Adolescent; PSQ, Perceived Stress Questionnaire*.

** *p < 0.01*.

## Discussion

This study investigated the psychometric properties of the Norwegian version of the PSQ in a sample of 15-16 years old adolescents. This included evaluating the fit of previously proposed PSQ-models, both in females and males, tests of measurement invariance across gender and a preliminary evaluation of concurrent validity with respect to mindfulness (measured by the Norwegian version of the MAAS-A). In overall, the PSQ performance seemed to correspond to previous findings. Main findings suggested best model fit for the four-factor model of the PSQ, in both females and males, comprising the factors “worries”, “tension”, (lack of) “joy” and “demands”. Assuming that all fit indices values were acceptable for analyses, configural and metric invariance seemed supported (Table 2). The findings also provided preliminary support of concurrent validity with respect to mindfulness (Table 3).

The overall PSQ scale performance seemed to correspond to previous findings among young people, regarding both internal consistency (measured by Cronbach α) and mean scale scores (Levenstein et al., 1993; Fliege et al., 2005; Kocalevent et al., 2007; Montero-Marin et al., 2014a). The internal consistency of the MAAS-A also corresponded to previous findings (Brown et al., 2011; de Bruin et al., 2011). Generally, females presented with the highest item mean scores, except from on item 25 “You are lighthearted” (reversed index). Internal consistency (Cronbach α) and corrected item-scale correlations were also generally higher in females. However, females and males reported highest on the same three items; 1 “You feel rested”, 8 “You are tired” and 13 “You are full of energy”, all with reversed indexes. Further psychometric studies might investigate more systematically, which are the most informative and necessary PSQ items in adolescent samples, for example by use of Item Response Theory (IRT) models (Borders et al., 2017).

As presented in Table 2, the four-factor model of the PSQ showed best fit to the data in both females and males, also in the restricted models, and showed the best trade-off between model fit and model complexity. The four-factor model comprises the factors “worries” (item 9, 12, 15, 18, 22), “tension” (item 1, 10, 14, 26, 27), (lack of) “joy” (item 7, 13, 17, 21, 25), and “demands” (item 2, 4, 16, 29, 30). I females, the RMSEA was close to 0.06 and the SRMR was < 0.10 and hence met the proposed criteria for high model fit between the hypothesized model and the observed data (Hu and Bentler, 1999). The CFI and NNFI were > 0.90 and hence met the proposed cutoff criteria for rejection of adequate proportions of miss-specified models (Hu and Bentler, 1999). In general, these criteria also were met in males, but the NNFI-value was in the lower bound (Table 2). This might suggest less acceptable fit to the data from the male sample compared to the female sample. However, as Gu et al. (2016) remind us and Schermelleh-Engel et al. (2014) cautioned, cut-off criteria can be arbitrary. A model may provide a good fit to the data even when one or more fit indices suggest poor fit and vice versa, and high fit indices are often easier to obtain when correlations between variables are low, because the power to detect discrepancies from predictions are amplified (Schermelleh-Engel et al., 2014; Gu et al., 2016).

All tested PSQ-models showed greatest model fit (according to most fit indices) on data from the female sample (Table 2). The four-factor model showed the smallest differences in model fit between gender, and configural and metric invariances were supported according to the criteria (ΔCFI ≤ 0.01, ΔNNFI ≤ 0.02, and ΔRMSEA ≤ 0.015 in fit to the previous less stringent model), considering the NNFI-value in males acceptable for analyses (Table 2). Assuming this, the findings imply that females and males attribute the same meaning to the latent construct and interpret the items in the same way. However, when constraining both factor loadings and intercepts, the change in fit (with respect to ΔCFI and ΔNNFI) to the previous less stringent model exceeded the recommended range. Hence, full scalar invariance seemed not supported for the four-factor model. This might suggest that females and males use the response scales in dissimilar ways. The implication of this is that cross-group comparisons (between adolescent females and males) with respect to scores on the latent variables are uncertain and must be interpreted with caution (Lavoie and Douglas, 2012; van de Schoot et al., 2012).

It might be reasonable that the four-factor model of the PSQ, originally revealed and confirmed by Fliege et al. (2005), showed best fit to the data. This model was originally based on a comprehensive psychometric evaluation of the instrument, involving two studies (both exploratory and confirmatory) and large samples (Fliege et al., 2005). The samples were composed of both patients and healthy individuals, and multi-sample analyses supported measurement invariance across groups (Fliege et al., 2005). In contrast, the initial psychometric study, finding a seven-factor model of the instrument, was based on a relatively small sample and exploratory factor analyses only (Levenstein et al., 1993). Moreover, in the studies by Fliege et al. (2005), there was an approximately even distribution of females and males, also among the young adults (students). On the contrary, the study sample in Montero-Marin et al. (2014a), wherein a two-factor model of the PSQ was found (based on parallel analysis and confirmatory factor analyses on data form the same sample), comprised most females (70.7 %). Additionally, the sample was relatively small (*N* = 314), encompassing a considerable uniform group, i.e., Spanish dental students (Montero-Marin et al., 2014a).

The inter-scale correlations presented in Table 3 show lowest factor-scale correlation for (lack of) “joy.” As pointed out in Fliege et al. (2005), (lak of) “joy” is a positively formulated subscale (item 7, 21, 25, 13, 17, all with reversed indexes). Hence, this subscale's deviation from the total scale (PSQ) and the other factors might reflect an “item format solution” more than a “content solution.” The PSQ-MAAS-A correlations displayed in Table 3 show strongest associations with respect to overall PSQ and “tension” (item 8, 14, 1, 26, 27, 10). Previous studies on MAAS-A have reported strong (negative) associations between MAAS/ MAAS-A and psychological disorders (Cordon and Finney, 2008; Walsh et al., 2009; Brown et al., 2011; Kiken and Shook, 2014; Loucks et al., 2015). Additionally, prior studies on the PSQ have demonstrated strong associations between “tension” (as a factor of the PSQ) and psychological and psychosomatic symptoms (Levenstein et al., 1993; Fliege et al., 2005). Taken together, previous and present findings might imply that the (negative) association between “tension” and MAAS-A is influenced by their common sensitivity to psychological/psychosomatic symptoms.

### Clinical implications and further directions

According to this initial psychometric evaluation of the Norwegian PSQ, the instrument seems to be feasible in adolescents (aged 15–16 years). The study provides preliminary support for a four-factor model of the PSQ in Norwegian adolescents, addressing relevant aspects of perceived stress, i.e., worries, tension, (lack of) joy, and demands, which might be relevant in the development of stress-related health complaints and disorders. The instrument might be useful in psychosomatic research as well as in clinical and health promotion work. According to the preliminary analyses, females and males seem to attribute the same meaning to the latent construct and interpret the items in the same way (i.e., configural and metric invariance seemed supported). However, we suggest caution in comparing groups (females and males) straightforward on latent means, as adolescent females and males possibly use the response scale in dissimilar ways (i.e., full scalar invariance was not supported). Further studies are needed to confirm the findings from this study and to evaluate the instrument's potential to predict stress-related health complaints and disorders in adolescents.

### Strengths and limitations

The high response rate in this study is considered a great strength. Overall, the psychometric evaluation of the PSQ in Norwegian adolescents is regarded relevant, as the instrument might have the potential to predict psychosomatic symptoms (Levenstein et al., 1993, 2000; Sanz-Carrillo et al., 2002; Fliege et al., 2005). However, we also acknowledge several limitations. The findings are based on one sample, and further studies are probably needed to confirm these findings. We also recognize possible limitations to results due to pre-analytic assumptions, such as assuming one fit index value in the lower bound in males acceptable for analyses. Hence, the results should be critically appraised. Moreover, the preliminary evaluation of concurrent validity with respect to the MAAS-A was based on a subsample, and hence the findings from these analyses must be interpreted with caution (Table 3).

## Conclusion

This study evaluated the Norwegian version of the PSQ in an adolescent sample. The overall PSQ performance seemed to correspond to previous findings, and internal consistency was supported across gender. A four-factor model of the instrument showed best fit to the data in both females and males, comprising the factors “worries,” “tension,” (lack of) “joy,” and “demands.” Assuming that all fit indices were acceptable for analyses, configural and metric invariance of the four-factor model seemed supported. Full scalar invariance was not supported for the four-factor model, implying that cross-group comparisons (between females and males) on latent means are uncertain and must be interpreted with caution. Concurrent validity with respect to mindfulness (MAAS-A) was preliminary supported. Further studies might be needed to confirm the findings from this study and to evaluate the instrument's potential to predict stress-related disorders in adolescents.

## Author contributions

BØ has made substantial contributions to the conception and design of the work, the acquisition, analysis, and interpretation of data for the work, has drafted the work and revised it critically for important intellectual content, and approved the final version to be published. She agrees to be accountable for all aspects of the work in ensuring that questions related to the accuracy or integrity of any part of the work are appropriately investigated. HS has made contribution to the conception of the work and has approved the final version to be publised. MH has made contribution to the conception of the work, revised it and approved the final version to be published.

### Conflict of interest statement

The authors declare that the research was conducted in the absence of any commercial or financial relationships that could be construed as a potential conflict of interest.
